# Efficacy and Safety of PEGylated Recombinant Human Growth Hormone in Children With Growth Hormone Deficiency and Idiopathic Short Stature: A Real‐World Cohort Study

**DOI:** 10.1155/ije/2058589

**Published:** 2026-01-22

**Authors:** Liping Ge, Canmiao Zhao, Yang Yang, Lijun Xu, Meiyuan Sun, Yanfang Su, Fang Xu, Qi Huang, Na Tao

**Affiliations:** ^1^ Department of Endocrinology, Genetics and Metabolism, Kunming Children’s Hospital (Children’s Hospital Affiliated to Kunming Medical University), Kunming, 650031, Yunnan, China

**Keywords:** efficacy, growth hormone deficiency, idiopathic short stature, PEGylated recombinant human growth hormone, safety

## Abstract

**Background:**

Growth hormone deficiency (GHD) and idiopathic short stature (ISS) are common causes of short stature in children. In China, PEGylated recombinant human GH (PEG‐rhGH, Jintrolong) has been approved for the treatment of both conditions. This study aimed to evaluate the efficacy and safety of PEG‐rhGH in children diagnosed with GHD or ISS and to compare clinical outcomes between the two groups.

**Methods:**

This real‐world study included 91 treatment‐naïve children with short stature at Kunming Children’s Hospital between 2020 and 2021. Participants were categorized into the GHD group (*n* = 39) and the ISS group (*n* = 52) based on etiological diagnosis. All subjects received weekly subcutaneous PEG‐rhGH injections at an initial dose of 0.20 mg/kg/wk and were followed for 18 months. Growth‐related parameters were assessed throughout the study.

**Results:**

PEG‐rhGH treatment significantly improved height standard deviation score (Ht SDS) in both groups. In the GHD group, Ht SDS increased from −3.14 (−4.06, −2.02) at baseline to −1.53 (−1.98, −1.08) at Month 18 (*p* < 0.001), with a mean ΔHt SDS of 1.69 ± 0.98. The ISS group demonstrated an improvement from −3.33 ± 1.23 at baseline to −1.33 (−2.03, −0.92) at 18 months (*p* < 0.001), with a mean ΔHt SDS of 1.77 ± 1.06. No significant differences were identified between the groups regarding Ht SDS, ΔHt SDS, Insulin‐Like Growth Factor 1 SDS (IGF‐1 SDS), ΔIGF‐1 SDS, or height velocity (all *p* > 0.05). Thyroid function markers (T3, T4, FT3, FT4) and fasting plasma glucose levels remained within normal ranges throughout treatment, with no significant intergroup differences (all *p* > 0.05). No serious adverse events were observed.

**Conclusion:**

PEG‐rhGH effectively promoted height gain in children with GHD and ISS, with similar therapeutic efficacy in both groups. However, children with ISS required a longer duration to achieve catch‐up growth to normal height, potentially due to reduced GH sensitivity and a need for higher dosing. PEG‐rhGH was well tolerated, with a favorable safety profile in both cohorts.

## 1. Introduction

Growth hormone deficiency (GHD) and idiopathic short stature (ISS) are among the most common causes of pediatric short stature. In China, 3.2% of children with short stature have associated comorbidities [1]. The prevalence of isolated GHD is estimated at 1:4,000‐1:10,000 live births [2], with a cross‐sectional study reporting an incidence of 1:8,646 among children aged 9–16 years [3]. In contrast, ISS accounts for approximately 41% of pediatric short stature [4]. Daily administration of recombinant human growth hormone (rhGH) has been shown to effectively improve height outcomes in children with GHD [5] or ISS [6, 7]. However, there were long‐term treatment adherence and persistence key challenges, largely due to the burden of daily injections [8, 9]. These limitations have driven the development of long‐acting rhGH formulations designed to extend the hormone’s half‐life and reduce injection frequency [10]. Several long‐acting rhGH preparations are now available, including Jintrolong®, Eutropin Plus™, Sogroya®, and Skytrofa®. Among them, Jintrolong® was approved in China in 2014 and has since been indicated for GHD, ISS, and Turner syndrome. [11–13]. It should be noted that the approval of growth hormone therapy for ISS varies worldwide, whereas it is well recognized for other conditions such as SHOX gene mutations [14], which should be mentioned given their prevalence.

PEGylated rhGH (PEG‐rhGH) incorporates a branched polyethylene glycol molecule conjugated to the amino groups of rhGH [15]. This PEGylation technology enhances protein stability via shielding effects, prolongs systemic exposure by reducing renal clearance and receptor‐mediated binding, and minimizes nonspecific absorption and antigenic responses [16]. Consequently, PEG‐rhGH exhibits an extended half‐life and lower injection frequency, providing a promising alternative to daily rhGH therapy in managing pediatric short stature [17, 18]. To date, Jintrolong remains the only clinically validated PEG‐rhGH formulation [10]. Clinical studies have demonstrated that PEG‐rhGH exhibits superior efficacy compared to daily rhGH in improving height velocity (HV) and height standard deviation score (Ht SDS) among children with GHD [19]. Notably, recent meta‐analyses indicate that PEG‐rhGH outperforms other long‐acting rhGH formulations in improving both Ht SDS and HV, while also demonstrating a more favorable safety profile with fewer reported adverse events [20].

However, clinical evidence regarding the efficacy and safety of PEG‐rhGH in children with GHD and ISS remains limited, especially among Chinese children. Therefore, this study aimed to comprehensively evaluate the long‐term therapeutic efficacy and safety profile of PEG‐rhGH in children with GHD or ISS, with a specific focus on comparative outcomes between the two groups. The findings are expected to provide strengthened evidence to support clinical treatment strategies and individualized management in pediatric short stature.

## 2. Materials and Methods

### 2.1. Study Participants

A total of 91 children diagnosed with short stature were enrolled at the Department of Endocrinology, Kunming Children’s Hospital, between 2020 and 2021. The inclusion criteria were (1) height below the third percentile or −2 standard deviation (SD) for age, sex, and ethnicity‐matched reference populations; and (2) a confirmed diagnosis of either GHD [5] or ISS [21] per established diagnostic criteria. The exclusion criteria included the following: (1) hepatic or renal dysfunction, or glucose metabolism disorders; (2) coexisting thyroid dysfunction, congenital skeletal dysplasia, malignancies, or inherited metabolic diseases; and (3) any previous treatment with rhGH. The study protocol was approved by the Ethics Committee of Kunming Children’s Hospital (Approval No.: 2022‐03‐117‐K01). All participants′ guardians were fully informed of the study objectives and provided written informed consent.

### 2.2. Study Design

Based on etiological diagnosis, participants were stratified into two distinct cohorts: the GHD group (*n* = 39) and the ISS group (*n* = 52). All enrolled children were treatment‐naïve and initiated subcutaneous administration of PEG‐rhGH therapy (Jintrolong, GeneScience Pharmaceuticals, Changchun, China) at an initial dose of 0.2 mg/kg/wk. Comprehensive baseline and follow‐up data were systematically collected, including (1) growth parameters—standing height, body weight, body mass index (BMI), BMI standard deviation score (BMI SDS), target height, and bone age (BA); and (2) biochemical indicators: fasting plasma glucose (FPG), thyroid‐stimulating hormone (TSH), triiodothyronine (T3), thyroxine (T4), free T3 (FT3), free thyroxine (FT4), and Insulin‐Like Growth Factor‐1 (IGF‐1) levels.

**Figure 1:**
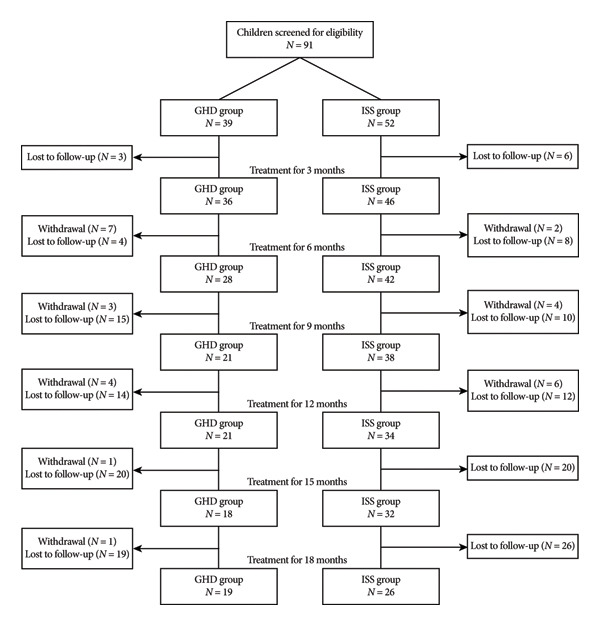
Flowchart of cases’ follow‐up in this study.

Follow‐up data were collected over an 18‐month period (Figure 1). Assessments were conducted at baseline and at 3, 6, 9, 12, 15, and 18 months of treatment, in accordance with the predefined data collection schedule. The analysis was performed from both longitudinal and horizontal dimensions. Longitudinal comparison examined within‐group changes in efficacy and safety indicators for GHD and children with ISS at baseline and after 3, 6, 9, 12, 15, and 18 months of treatment. Horizontal analysis compared between‐group variations in these indicators at each time point. The efficacy indicators included the following: (1) Ht SDS and its change (ΔHt SDS); (2) IGF‐1 standard deviation score and its change (ΔIGF‐1 SDS); and (3) HV. The safety indicators comprised thyroid function makers (TSH, T3, T4, FT3, FT4) and FPG levels.

### 2.3. Measurement Methods

Serum IGF‐1 levels were measured in all participants using a Siemens Immulite 2000 automated chemiluminescence immunoanalyzer. For all children diagnosed with GHD, the diagnosis was confirmed by GH stimulation testing in accordance with Chinese guidelines, with a peak growth hormone level below 10 ng/mL in both tests [5]. The GST was evaluated via two stimulation tests: L‐dopa (10 mg/kg, maximum 500 mg) and 10% arginine (2 mL/kg, equivalent to 0.5 g/kg, maximum 30 g). GH concentrations were assessed at 0, 30‐, 60‐, 90‐, and 120 min postadministration. FPG levels were determined from fingertip blood collected under sterile conditions after an overnight fast of 8–12 h.

All children diagnosed with ISS in this study underwent comprehensive evaluations to exclude other possible causes of short stature [21]. These included detailed assessments of growth and developmental history, family history, and physical examination. Routine laboratory tests (such as complete blood count, biochemical profile, and electrolytes) were performed to exclude chronic diseases and malnutrition. Endocrine evaluations included thyroid function tests, serum IGF‐1 levels, and, when necessary, GH stimulation tests and pituitary MRI to rule out endocrine disorders. BA assessment was performed for all participants. In addition, genetic testing (such as karyotype analysis) was undertaken when clinically indicated to exclude known genetic syndromes. Only children with no identifiable underlying cause after these evaluations were diagnosed with ISS and included in the study.

Efficacy outcomes were defined as follows: Height was standardized using the Height Standardized Growth Charts for Chinese Children and Adolescents aged 0–18 years [22]. Ht SDS was calculated as (measured height – mean height of age‐ and sex‐matched healthy children)/(SD of height in age‐ and sex‐matched healthy children); ΔHt SDS was defined as (Ht SDS at follow‐up – Ht SDS at baseline). Similarly, IGF‐1 SDS was calculated [23] as (measured IGF‐1 – mean IGF‐1 of age‐ and sex‐matched healthy children)/(SD of IGF‐1 in age‐ and sex‐matched healthy children), and its change (ΔIGF‐1 SDS) was defined as (IGF‐1 SDS at follow‐up − IGF‐1 SDS at baseline). HV was calculated as follows: HV (cm/year) = 12 × (height at follow‐up − height at baseline)/follow‐up duration (months).

### 2.4. Statistical Analysis

All statistical analyses were performed using IBM SPSS Statistics Version 27, and data visualization was conducted in Origin 2024. Categorical variables were presented as counts and percentages (*n*, %), and continuous variables were summarized using mean ± SD ($\overset{¯}{x}$ ± SD) for normally distributed data or median with interquartile range [M (P25, P75)] for non‐normally distributed data. Baseline comparisons between groups were conducted using independent samples *t* tests for normally distributed variables, Mann–Whitney *U* tests for non‐normally distributed variables, and chi‐square tests for categorical variables. Efficacy and safety outcomes were analyzed using generalized estimating equations, with post hoc comparisons adjusted by the Bonferroni correction. All statistical tests were two‐tailed, and a *p* value < 0.05 was considered statistically significant.

## 3. Results

### 3.1. Baseline Characteristics

At baseline, the median age was 5.79 years in the GHD group and 6.06 years in the ISS group. The GHD group included 22 males (56.41%) and 17 females (43.59%), while the ISS group included 28 males (53.85%) and 24 females (46.15%). No significant differences were observed between the groups in age, BA, height, target height, BMI, BMI SDS, Ht SDS, IGF‐1, or IGF‐1 SDS (all *p* > 0.05). The GHD group had a significantly lower BA/chronological age (BA/CA) ratio than the ISS group (*p* = 0.012), indicating a more pronounced BA delay. Baseline peak GH levels were also significantly lower in the GHD group compared with the ISS group (*p* < 0.001). A detailed summary of baseline characteristics is provided in Table 1.

**Table 1:** Baseline characteristics of enrolled children.

Characteristics	GHD group (*n* = 39)	ISS group (*n* = 52)	*t*/*χ* ^2^/*Z*	*p* value
Sex				
Male, *n* (%)	22 (56.41)	28 (53.85)	0.059	0.809
Female, *n* (%)	17 (43.59)	24 (46.15)		
Age, y	5.79 (4.76, 9.04)	6.06 (5.27, 8.35)	0.938	0.348
BA, y	3.50 (2.50, 7.00)	5.00 (3.88, 7.13)	1.632	0.103
BA/CA	0.63 (0.50, 0.77)	0.74 ± 0.16	2.512	**0.012**
Height, cm	105.20 (97.00, 120.30)	106.75 (102.13, 118.88)	0.890	0.373
Target height, cm	163.43 ± 7.07	161.50 (155.13, 168.50)	1.203	0.229
Ht SDS	−3.14 (−4.06, −2.02)	−3.33 ± 1.23	0.413	0.680
IGF‐1, ng/mL	134.97 ± 68.04	151.75 ± 57.12	1.257	0.212
IGF‐1 SDS	−1.26 ± 0.82	−1.15 (−1.49, −0.56)	1.828	0.068
Peak GH levels, μg/L	6.82 ± 1.92	13.92 (12.10, 16.94)	7.801	**< 0.001**
BMI	14.69 (14.01, 15.94)	14.71 ± 1.11	0.714	0.475
BMI SDS	−0.56 (−1.27, 0.35)	−0.65 (−1.18, −0.35)	0.874	0.382

*Note:* Unless otherwise specified, continuous variables are presented as $\overset{¯}{x}$±SD (normally distributed) or M (P25, P75) (non‐normally distributed). The bold values indicate statistically significant results (*p* < 0.05). BA/CA: bone age/chronological age ratio; Ht SDS: height standard deviation score; IGF‐1: Insulin‐Like Growth Factor‐1; IGF‐1 SDS: Insulin‐Like Growth Factor‐1 SDS.

Abbreviations: BA, bone age; BMI, body mass index; BMI SDS, body mass index SDS; GH, growth hormone.

### 3.2. Height Growth During PEG‐rhGH Therapy

Following PEG‐rhGH treatment, the GHD group demonstrated a significant increase in Ht SDS, improving from −3.14 (−4.06, −2.02) at baseline to −1.53 (−1.98, −1.08) at 18 months (*p* < 0.001). All follow‐up time points showed statistically significant increases compared to baseline (all *p* < 0.05). Similarly, the ISS group showed a marked improvement in Ht SDS, rising from −3.33 ± 1.23 at baseline to −1.33 (−2.03, −0.92) at 18 months (*p* < 0.001), with each follow‐up measurement significantly higher than baseline (all *p* < 0.05). There was no significant difference in Ht SDS between groups over the treatment period (Wald *χ* ^2^ = 0.122, *p* = 0.727), indicating comparable growth responses to PEG‐rhGH in both groups. As illustrated in Figure 2(a), median Ht SDS in both groups exhibited a positive correlation with treatment duration. The GHD group reached the threshold for normal height (≥ −2 SD) at 9 months, whereas the ISS group achieved this milestone at 12 months, suggesting a potentially faster catch‐up growth trajectory in children with GHD.

Figure 2Violin plots of height growth in children with GHD and ISS during PEG‐rhGH therapy.

**Figure:**
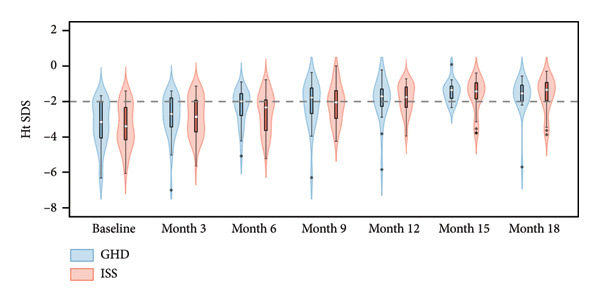
(a)

**Figure:**
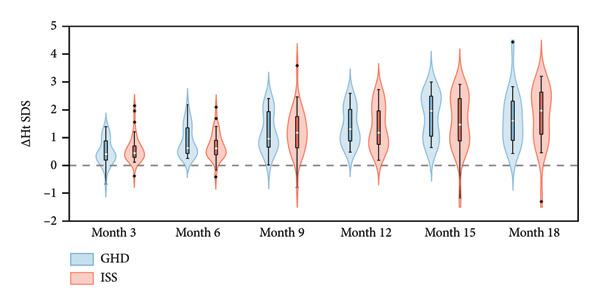
(b)

**Figure:**
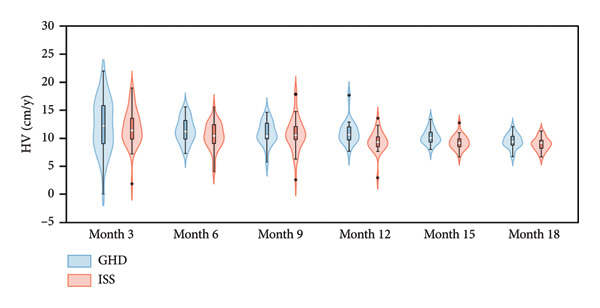
(c)

Both groups exhibited significantly greater ΔHt SDS at 6, 9, 12, 15, and 18 months of treatment compared to the 3‐month time point (all *p* < 0.05). No significant between‐group variations in ΔHt SDS was observed (Wald *χ* ^2^ = 0.075, *p* = 0.784), indicating a comparable magnitude of height improvement between the two groups throughout PEG‐rhGH therapy. As shown in Figure 2(b), mean ΔHt SDS was positively correlated with treatment duration in both cohorts.

Both groups showed a similar gradual decline in HV with prolonged PEG‐rhGH therapy. Nevertheless, a median HV of approximately 9 cm/year was maintained at 18 months of therapy, with no statistically significant difference between the ISS and GHD groups (Table 2).

**Table 2:** Height growth in the GHD group and ISS group during PEG‐rhGH therapy.

	GHD group	ISS group	Wald *χ* ^2^(*p*)		
			Group effect	Time effect	Interaction
Ht SDS			0.122 (0.727)	200.476 (**< 0.001**)	3.666 (0.722)
Baseline	−3.14 (−4.06, −2.02)	−3.33 ± 1.23			
Month 3	−2.70 (−3.46, −1.77)^a^	−2.84 ± 1.14^a^			
Month 6	−1.98 (−2.82, −1.55)^a^	−2.63 ± 1.13^a^			
Month 9	−1.77 (−2.72, −1.22)^a^	−2.11 ± 1.06^abc^			
Month 12	−1.70 (−2.29, −1.24)^ab^	−1.74 (−2.39, −1.12)^abc^			
Month 15	−1.42 ± 0.61^abc^	−1.42 (−1.90, −0.92)^abc^			
Month 18	−1.53 (−1.98, −1.08)^a^	−1.33 (−2.03, −0.92)^abc^			
ΔHt SDS			0.075 (0.784)	112.081 (**< 0.001**)	5.588 (0.348)
Month 3	0.49 ± 0.47	0.45 (0.29, 0.70)			
Month 6	0.63 (0.43, 1.40)^b^	0.69 ± 0.48^**b**^			
Month 9	1.22 ± 0.71^bc^	1.22 ± 0.80^bc^			
Month 12	1.43 ± 0.67^bc^	1.39 ± 0.77^bc^			
Month 15	1.78 ± 0.81^bc^	1.57 ± 0.99^bc^			
Month 18	1.69 ± 0.98^bcd^	1.77 ± 1.06^bcd^			
HV			3.364 (0.067)	65.640 (**< 0.001**)	5.918 (0.314)
Month 3	11.62 ± 4.36	11.94 ± 2.23			
Month 6	11.70 ± 1.60	10.22 ± 1.62			
Month 9	10.59 ± 1.31	10.57 (9.90, 12.85)			
Month 12	10.43 ± 1.05	9.28 (8.07, 10.14)^b^			
Month 15	9.72 ± 1.34	8.86 ± 1.10^b^			
Month 18	9.15 ± 1.15^bcde^	8.96 ± 1.12^bcd^			

*Note:* Unless otherwise specified, continuous variables are presented as $\overset{¯}{x}\pm \text{SD}$ (normally distributed) or M (P25, P75) (non‐normally distributed). The bold values indicate statistically significant results (*p* < 0.05).

^a^ *p* < 0.05 vs baseline.

^b^ *p* < 0.05 vs month 3.

^c^ *p* < 0.05 vs month 6.

^d^ *p* < 0.05 vs month 9.

^e^ *p* < 0.05 vs month 12.

### 3.3. IGF‐1 Levels During PEG‐rhGH Therapy

The GHD group demonstrated significant increases in IGF‐1 SDS at all follow‐up time points compared to baseline (all *p* < 0.05). Similarly, the ISS group showed significantly higher IGF‐1 SDS at 3, 6, 9, 12, and 15 months relative to baseline (all *p* < 0.05). No significant between‐group variations in IGF‐1 SDS was observed between the two groups (Wald *χ* ^2^ = 3.803, *p* = 0.051), indicating comparable IGF‐1 responses to PEG‐rhGH therapy. As illustrated in Figure 3(a), median IGF‐1 SDS in both groups remained within ±1 SD during PEG‐rhGH therapy.

Figure 3Violin plots of IGF‐1 level changes in children with GHD and ISS during PEG‐rhGH therapy.

**Figure:**
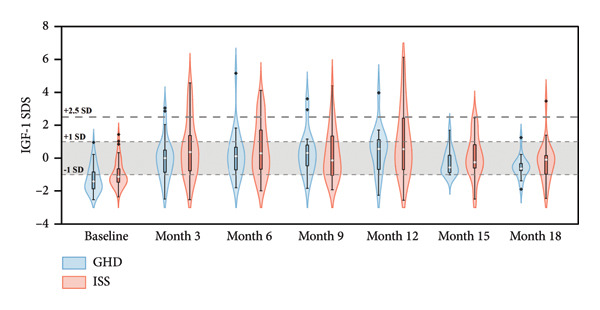
(a)

**Figure:**
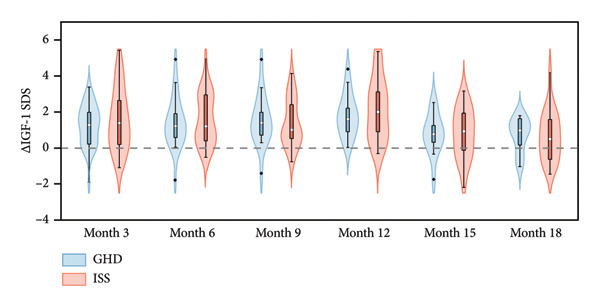
(b)

No significant between‐group differences were found in ΔIGF‐1 SDS (Wald *χ* ^2^ = 0.551, *p* = 0.458), suggesting comparable IGF‐1 dynamics across both groups during PEG‐rhGH therapy. However, both groups exhibited a significant decline in ΔIGF‐1 SDS at 15 and 18 months compared to the 12‐month time point (all *p* < 0.05) (Table 3).

**Table 3:** Changes in IGF‐1 levels in children with GHD and ISS during PEG‐rhGH therapy.

	GHD group	ISS group	Wald *χ* ^2^(*p*)		
			Group effect	Time effect	Interaction
IGF‐1 SDS			3.803 (0.051)	203.353 (**< 0.001**)	3.036 (0.804)
Baseline	−1.26 ± 0.82	−1.15 (−1.49, −0.56)			
Month 3	−0.04 ± 1.24^a^	0.58 ± 1.76^a^			
Month 6	0.13 (−0.72, 0.69)^a^	0.59 ± 1.65^a^			
Month 9	0.30 ± 1.33^a^	0.25 ± 1.53^a^			
Month 12	0.49 ± 1.39^a^	1.07 ± 2.14^a^			
Month 15	−0.55 (−0.87, 0.18)^a^	0.10 ± 1.30^a^			
Month 18	−0.56 ± 0.67^ae^	−0.09 (−0.97, 0.18)			
ΔIGF‐1 SDS			0.551 (0.458)	24.559 (**< 0.001**)	1.694 (0.890)
Month 3	1.24 ± 1.14	1.58 ± 1.72			
Month 6	1.41 ± 1.38	1.61 ± 1.41			
Month 9	1.54 ± 1.39	1.40 ± 1.31			
Month 12	1.82 ± 1.16	2.17 ± 1.72			
Month 15	0.80 ± 1.13^e^	0.81 ± 1.40^e^			
Month 18	0.99 (−0.08, 1.63)^ce^	0.67 ± 1.37^ce^			

*Note:* Unless otherwise specified, continuous variables are presented as $\overset{¯}{x}\pm \text{SD}$ (normally distributed) or M (P25, P75) (non‐normally distributed). The bold values indicate statistically significant results (*p* < 0.05).

^a^ *p* < 0.05 vs baseline.

^b^ *p* < 0.05 vs month 3.

^c^ *p* < 0.05 vs month 6.

^d^ *p* < 0.05 vs month 9.

^e^ *p* < 0.05 vs month 12.

### 3.4. Safety Indicators During PEG‐rhGH Therapy

In the GHD group, levels of T3, T4, and FT4 remained stable throughout the treatment period (all *p* > 0.05), while TSH level significantly reduced at 3 months versus baseline (*p* = 0.027), and FT3 significantly decreased at 18 months compared to 3 months (*p* = 0.003). In the ISS group, TSH, T3, T4, and FT4 levels remained stable (all *p* > 0.05), whereas FT3 levels at 15 months were significantly lower than at 3 and 9 months (both *p* < 0.05). No significant between‐group differences were observed for any thyroid function markers (all *p* > 0.05) (Table 4).

**Table 4:** Changes in safety indicators in children with GHD and ISS during PEG‐rhGH therapy.

	GHD group	ISS group	Wald *χ* ^2^(*p*)		
			Group effect	Time effect	Interaction
TSH			1.766 (0.184)	17.586 (**0.007**)	2.676 (0.848)
Baseline	3.01 ± 1.36	2.55 (1.80, 3.48)			
Month 3	2.52 ± 1.32^a^	2.34 (1.55, 3.13)			
Month 6	2.90 (2.13, 4.57)	2.57 ± 1.19			
Month 9	3.01 ± 1.66	2.61 ± 2.30			
Month 12	2.98 ± 1.99	2.03 (1.63, 2.79)			
Month 15	2.51 ± 1.06	1.93 (1.55, 2.82)			
Month 18	2.42 ± 1.20	1.82 (1.43, 2.56)			
T3			1.097 (0.295)	6.661 (0.353)	6.940 (0.326)
Baseline	2.44 ± 0.41	2.32 ± 0.39			
Month 3	2.57 ± 0.35	2.63 (2.30, 2.89)			
Month 6	2.59 ± 0.30	2.50 ± 0.46			
Month 9	2.38 ± 0.39	2.60 (2.30, 2.84)			
Month 12	2.37 ± 0.36	2.34 ± 0.40			
Month 15	2.48 ± 0.28	2.33 ± 0.33			
Month 18	2.46 ± 0.30	2.41 ± 0.46			
T4			1.091 (0.296)	6.272 (0.393)	7.798 (0.253)
Baseline	124.72 ± 20.47	118.78 ± 21.57			
Month 3	122.84 ± 19.78	120.45 ± 24.53			
Month 6	116.57 ± 20.57	118.20 ± 21.98			
Month 9	112.37 ± 21.77	121.25 ± 23.32			
Month 12	120.91 ± 18.73	120.57 ± 23.45			
Month 15	119.40 (98.10, 132.90)	117.44 ± 16.73			
Month 18	121.50 ± 17.06	113.29 ± 26.64			
FT3			0.048 (0.826)	25.203 (**< 0.001**)	3.473 (0.748)
Baseline	6.43 (6.08, 6.86)	6.65 (6.21, 7.01)			
Month 3	6.67 ± 0.73	6.81 (6.37, 7.72)			
Month 6	6.81 ± 0.80	6.94 ± 0.86			
Month 9	6.48 (6.19, 7.32)	7.09 (6.50, 7.76)			
Month 12	6.39 ± 0.94	6.48 (6.04, 7.00)			
Month 15	6.64 ± 0.83	6.32 ± 0.63^bc^			
Month 18	5.93 ± 0.80^b^	6.45 ± 0.75			
FT4			3.535 (0.060)	16.417 (**0.012**)	3.666 (0.722)
Baseline	19.38 (17.83, 20.44)	19.68 ± 1.35			
Month 3	18.88 ± 2.23	19.07 ± 1.76			
Month 6	18.27 ± 2.37	19.13 (17.95, 20.60)			
Month 9	18.12 ± 0.61	19.27 ± 1.59			
Month 12	19.40 ± 1.58	19.62 (18.26, 21.08)			
Month 15	18.87 ± 1.99	20.46 (19.01, 21.32)			
Month 18	19.70 ± 2.33	20.03 ± 1.78			
FPG			0.258 (0.612)	37.369 (**< 0.001**)	12.486 (0.052)
Baseline	3.93 ± 0.52	4.13 ± 0.48			
Month 3	4.27 ± 0.51	4.29 ± 0.58			
Month 6	4.44 ± 0.43^a^	4.30 ± 0.53			
Month 9	4.50 ± 0.41^a^	4.30 (4.00, 4.50)			
Month 12	4.42 ± 0.59	4.41 ± 0.51			
Month 15	4.40 (4.15, 4.56)^a^	4.50 (4.05, 4.70)			
Month 18	4.30 (4.03, 4.65)	4.58 ± 0.44^a^			

*Note:* Unless otherwise specified, continuous variables are presented as *x* ± SD (normally distributed) or M (P25, P75) (non‐normally distributed). The bold values indicate statistically significant results (*p* < 0.05).

^a^ *p* < 0.05 vs baseline.

^b^ *p* < 0.05 vs month 3.

^c^ *p* < 0.05 vs month 9.

Regarding glucose metabolism, the GHD group exhibited significantly elevated FPG levels at 6, 9, and 15 months compared to baseline (all *p* < 0.05). In the ISS group, FPG levels were significantly increased at 18 months relative to baseline (*p* = 0.002). However, no significant differences in FPG levels were detected between the two groups (Wald *χ* ^2^ = 0.258, *p* = 0.612), indicating comparable FPG responses to PEG‐rhGH therapy.

## 4. Discussion

This study presents the first comparative evaluation of medium‐to‐long‐term outcomes of PEG‐rhGH therapy in children with GHD and ISS. Both groups demonstrated significant improvements in Ht SDS and IGF‐1 SDS from baseline (all *p* < 0.05), and no significant differences were detected between the groups across any efficacy parameters (all *p* > 0.05). These results indicate that PEG‐rhGH confers comparable height‐promoting effects in pediatric patients with either GHD or ISS.

In the present study, the GHD group had a baseline median Ht SDS of −3.14 and achieved a mean ΔHt SDS of +1.69 after 18 months of treatment. In comparison, a multicenter study by Luo et al. [18] reported a baseline mean Ht SDS of −2.36 and mean ΔHt SDS of +1.63 after 36 months of PEG‐rhGH therapy (0.2 mg/kg/wk) in children with GHD. Among children with ISS, our cohort demonstrated a baseline mean Ht SDS of −3.33 and a mean ΔHt SDS of +1.77 at 18 months, while Luo et al. [17] reported a baseline mean Ht SDS of −2.53 and mean ΔHt SDS of +0.98 at 18 months. Furthermore, Xie et al. [24] reported a mean ΔHt SDS of +1.06 at 12 months and +1.65 at 24 months in children with ISS under equivalent dosing of PEG‐rhGH, starting from a baseline mean Ht SDS of −2.38. Compared with these studies, our cohort demonstrated more pronounced height improvement and earlier growth benefits. This may be attributable to the lower baseline Ht SDS observed in our study population, likely reflecting regional differences in pediatric growth patterns across China. Supporting this, a nationwide survey by Zhang et al. [25] involving 110,499 Chinese children found that the prevalence of moderate‐to‐severe stunting (Z score < −2.5) was significantly higher in southern regions (23.5%, including Kunming) than in northern (20.0%) and central China (16.5%) (*p* = 0.005). Notably, Kunming ranked second highest in the prevalence of severe stunting (Z score < 3.0) among the nine cities surveyed.

In this study, all children received an initial PEG‐rhGH dose of 0.2 mg/kg/wk and achieved comparable height gains (ΔHt SDS: 1.69 ± 0.98 vs. 1.77 ± 1.06, *p* > 0.05). However, the ISS group exhibited a 3‐month delay in reaching catch‐up growth (defined as Ht SDS > −2 SD) compared to the GHD group (12 vs. 9 months), which is potentially attributable to the lack of a higher initial PEG‐rhGH dose in the ISS group. The optimal rhGH dosing strategy for ISS remains an unresolved clinical question, particularly in light of partial GH resistance and possible GH insensitivity [26]. Some studies have suggested [27] that dosing regimens designed for GHD may be insufficient to achieve maximal height outcomes in ISS children. Supporting this, a Multicenter Phase II study of PEG‐rhGH in children with ISS [17] demonstrated dose‐dependent improvements in Ht SDS, HV, and IGF‐1 SDS, indicating enhanced responsiveness at higher doses. Furthermore, previous studies [28] found that children with ISS often require higher rhGH doses than children with GHD to achieve equivalent IGF‐1 SDS levels, underscoring the need for specific management strategies in the ISS population during PEG‐rhGH therapy. In our cohort, baseline assessments revealed that children in the GHD group had significant BA delay and lower peak GH levels compared to those in the ISS group, suggesting greater treatment sensitivity. Donbaloğlu et al. [29] similarly reported that children with GHD with baseline peak GH < 3 μg/L experienced significantly greater improvement in Ht SDS during the first two years of treatment compared to those with higher GH peaks (*p* = 0.040, 0.033). These findings suggest that more severe GH deficiency is associated with better responsiveness to rhGH. Future studies may consider stratifying PEG‐rhGH dosing based on baseline GH peaks to guide personalized treatment approaches in children with GHD.

In this study, most children exhibited increased IGF‐1 SDS levels during PEG‐rhGH therapy while remaining within the recommended upper limit of +2.5 SD for age‐ and sex‐matched norms. Current rhGH treatment guidelines [30] recommend dose reduction or discontinuation when serum IGF‐1 levels persistently exceed +2.5 SD. However, these recommendations were developed for daily rhGH regimens; with PEG‐rhGH, higher peak IGF‐1 levels may be necessary to achieve clinical efficacy, although the implications of transient IGF‐1 elevation remain unclear [10]. Notably, children with ISS may display partial IGF‐1 insensitivity [26], which could limit the utility of IGF‐1‐guided dose adjustments in this population. In our cohort, the few children with IGF‐1 levels > +2.5 SD returned to within the normal range following dose adjustment. At 15 and 18 months, IGF‐1 SDS levels remained higher than baseline, although ΔIGF‐1 SDS declined significantly compared with 12 months, particularly among children with ISS (Figure 3(b)). This may reflect insufficient dose escalation during the second year of treatment. Xie et al. [24] reported better IGF‐1 responses (ΔIGF‐1 SDS = 1.51 ± 1.08 at 2 years) with a more aggressive dosing strategy (initial 0.2 mg/kg/wk, maximum 0.3 mg/kg/wk), compared to the conservative dosing used in our study (0.18–0.22 mg/kg/wk). Additionally, an increasing number of treatment discontinuations occurred after the first year, which may have further influenced these findings.

Given the extended pharmacodynamic profile of long‐acting rhGH, its safety has become a critical focus of ongoing research. In our study, thyroid function markers remained within normal ranges in the majority of children throughout the course of PEG‐rhGH therapy, with no significant differences observed between groups. However, significant reductions in TSH and FT3 were noted at specific time points compared to baseline. These findings are consistent with those of Du et al. [31], who reported a significant decrease in TSH in children with GHD after six months of PEG‐rhGH therapy (*p* = 0.003), but contrast with the results from Hou et al. [18], who observed no significant fluctuations in T3, T4, or TSH levels (all *p* > 0.05). Notably, although previous studies [27, 32, 33] have reported cases of clinical and subclinical hypothyroidism during PEG‐rhGH therapy, none were observed in our 18‐month follow‐up. However, given that subclinical or clinically silent hypothyroidism may develop gradually over longer treatment durations, the possibility of delayed thyroid dysfunction cannot be completely ruled out. Therefore, continued long‐term thyroid monitoring remains necessary. So far, the mechanism by which PEG‐rhGH may influence thyroid function remains unclear, and no conclusive evidence has established a causal relationship between PEG‐rhGH use and thyroid dysfunction in children with GHD or ISS. Considering existing evidence, routine monitoring of thyroid parameters during PEG‐rhGH therapy is advisable in clinical practice. Moreover, there is a clear need for long‐term safety studies specifically focused on the thyroid‐related effects of PEG‐rhGH in pediatric populations with short stature, as its impact could be more complex and far‐reaching than currently understood. With regard to metabolic safety, although transient FPG elevation was observed at certain follow‐up visits, all values remained within the normoglycemic range. These results are consistent with previous studies [11, 17, 34], which found no significant effect of PEG‐rhGH therapy on glucose metabolism. The overall safety profile of PEG‐rhGH in this study was favorable, with only mild adverse events reported and no serious events observed.

This study has several limitations. First, the sample size was relatively small, and the enrolled participants covered a broad age range, with some having entered puberty. Therefore, the potential influence of pubertal development, particularly the role of estrogen in female patients, on height outcomes during PEG‐rhGH therapy cannot be fully excluded. Second, the relatively high dropout rate during follow‐up may have introduced bias, particularly affecting data integrity and reducing statistical power at the 16‐ and 18‐month time points.

## 5. Conclusion

This study demonstrates that PEG‐rhGH therapy produces significant and comparable height improvements in children with GHD and ISS. However, children with ISS required a longer duration to achieve catch‐up growth to normal height compared to those with GHD, possibly due to impaired GH sensitivity and a higher dosing requirement. The treatment exhibited a favorable safety profile in both groups. Mild reductions in TSH and FT3, along with elevated FPG, were observed in some patients, underscoring the need for regular monitoring of thyroid function and glucose metabolism during PEG‐rhGH therapy. Overall, these findings provide additional evidence supporting the clinical use of PEG‐rhGH in the management of pediatric short stature.

**Nomenclature**
GH: Growth hormone
GHD: Growth hormone deficiency
ISS: Idiopathic short stature
rhGH: Recombinant human growth hormone
PEG‐rhGH: PEGylated recombinant human growth hormone
HV: Height velocity
BA: Bone age
BA/CA: Bone age/chronological age
Ht SDS: Height standard deviation score
BMI: Body mass index
BMI SDS: BMI standard deviation score
FPG: Fasting plasma glucose
TSH: Thyroid‐stimulating hormone
T3: Triiodothyronine
T4: Thyroxine
FT3: Free triiodothyronine
FT4: Free thyroxine
IGF‐1: Insulin‐like growth factor‐1
ΔHt SDS: Change of Ht SDS
IGF‐1 SDS: IGF‐1 standard deviation score
ΔIGF‐1 SDS: Change of IGF‐1 SDS
SD: Standard deviations

## Ethics Statement

This study was approved by the Research Ethics Committees of Kunming Children’s Hospital (Approval No.: 2022‐03–117‐K01). No experimental interventions were conducted. All procedures performed were in accordance with the Declaration of Helsinki (revised in 2013).

## Conflicts of Interest

The authors declare no conflicts of interest.

## Author Contributions

Na Tao was responsible for research supervision, guidance, manuscript review, and revision. Liping Ge carried out research conception and design, data analysis, and initial manuscript drafting. Fang Xu, Yanfang Su, and Yang Yang conducted clinical data collection. Canmiao Zhao and Meiyuan Sun were responsible for data analysis. Lijun Xu and Qi Huang took charge of data integration and literature retrieval. Canmiao Zhao also contributed to initial manuscript drafting. All authors revised the manuscript critically and provided significant academic input. Na Tao and Zhao Canmiao contributed equally to this work.

## Funding

This study was funded by Kunming Municipal Health Science and Technology Talent Cultivation Project—Reserve Talent Cultivation Program in Medical Science and Technology, Kunming Children’s Hospital.

## Data Availability

The data that support the findings of this study are available from the corresponding author upon reasonable request.
